# Supplementary material for: scRepli-RamDA-seq: a multi-omics technology enabling the analysis of gene expression dynamics during S-phase
Source: Nat Commun. 2025 Dec 15;16:10902. doi: 10.1038/s41467-025-64688-1 (PMC12705686; doi:10.1038/s41467-025-64688-1)
Supplement: Supplementary file 1 — Supplementary Information [file 41467_2025_64688_MOESM1_ESM.pdf]

Supplementary Information

scRepli-RamDA-seq: a multi-omics technology enabling the analysis of gene expression dynamics during S-phase

Rawin Poonperm[1,2,*], Taiki Yoneda[2,*], Taito Imada[2], Saori Takahashi[1], Takako Ichinose[1], Hisashi Miura[1], Tetsutaro Hayashi[3], Mariko Kuse[3], Mika Yoshimura[3], Koji Nagao[4], Chikashi Obuse[4], Itoshi Nikaido[3,5] ✉, Ichiro Hiratani[1] ✉, Shin-ichiro Takebayashi[2] ✉

[1]Laboratory for Developmental Epigenetics, RIKEN Center for Biosystems Dynamics Research (BDR), Kobe, Japan. [2]Laboratory of Molecular and Cellular Biology, Graduate School of Bioresources, Mie University, Tsu, Japan. [3]Omics AI Research Team, Advanced General Intelligence in Science Program (AGIS), TRIP Headquarters, RIKEN, Wako, Saitama, Japan. [4]Department of Biological Sciences, Graduate School of Science, The University of Osaka, Toyonaka, Japan. [5]Department of Functional Genome Informatics, Division of Biological Data Science, Medical Research Laboratory (MRL), Institute of Integrated Research (IIR), Institute of Science Tokyo, Tokyo, Japan. [*]These authors contributed equally.

✉ e-mail: itoshi.nikaido@riken.jp; ichiro.hiratani@riken.jp; stake@bio.mie-u.ac.jp

List of Supplementary Information

- Supplementary Figures 1 to 15
- Supplementary Notes 1 to 2
- Supplementary Table 1
- Supplementary References

Supplementary Figures

Supplementary Figure 1

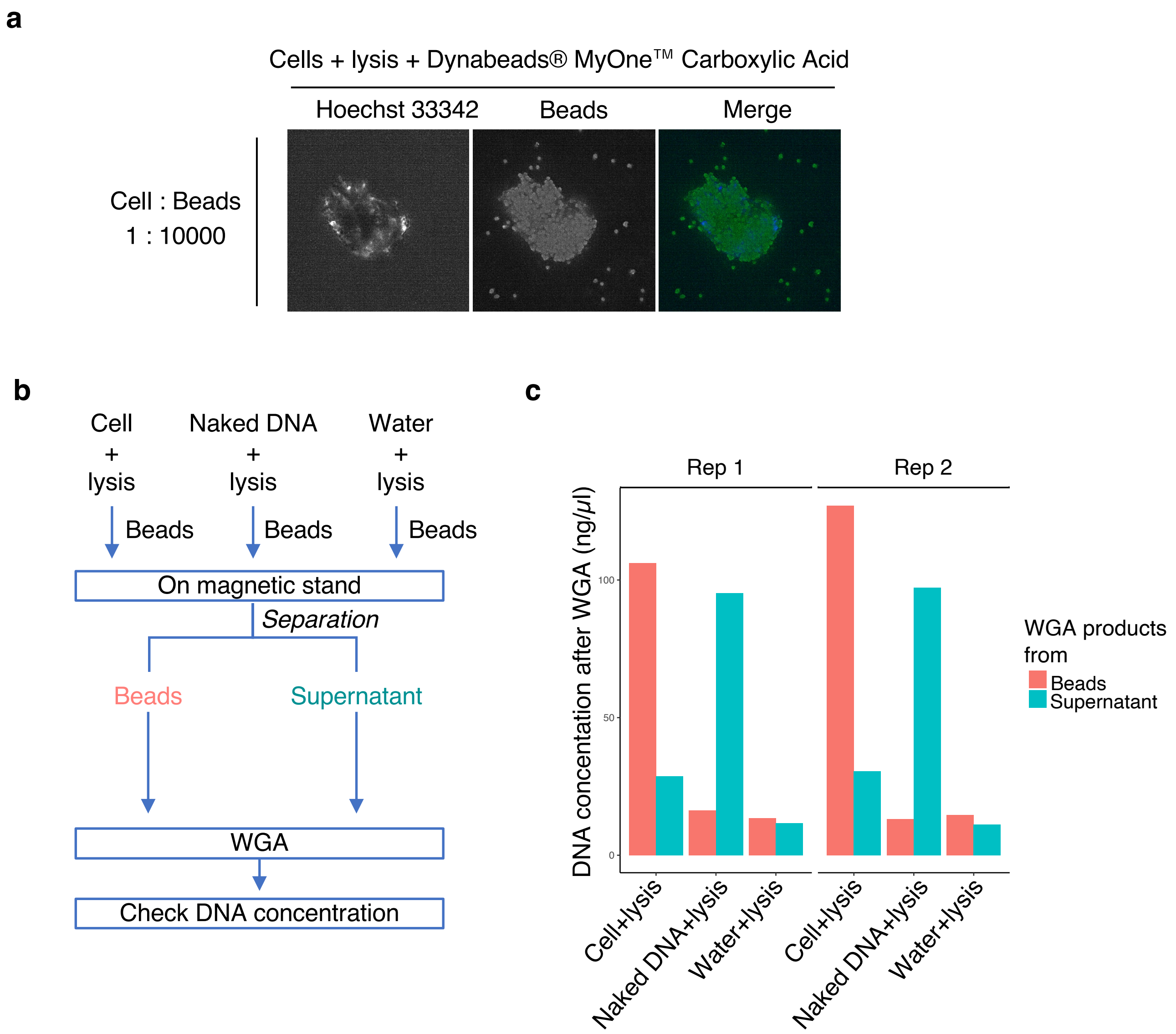

Supplementary Figure 1. Dynabeads® MyOne™ Carboxylic Acid captures genomic DNA but no naked DNA.

a) Mouse embryonic stem cells (mESCs) were stained with the DNA-binding dye Hoechst 33342, then lysed in RamDA lysis buffer containing Dynabeads® MyOne™ Carboxylic Acid (beads) at a ratio of 1 cell per 10,000 beads. The magnetic beads were separated and fixed with 2% paraformaldehyde. After washing, the magnetic suspension was placed on a glass slide. Under fluorescence microscopy, the beads exhibited autofluorescence when excited at a 488 nm wavelength[1]. We observed that the beads (green) clustered around Hoechst 33342-

positive particles (blue), suggesting that the beads likely captured genomic DNA. **b)** To test whether the beads could directly capture naked DNA, we performed an experiment by mixing either cells or naked DNA isolated from mouse cells with RamDA lysis buffer, followed by the addition of beads. Note that we adjusted equal amounts of DNA from cells and naked DNA. After incubating the mixture for 5 minutes, we separated the beads from the supernatant and used both for whole-genome amplification (WGA). We then purified the WGA products and measured DNA concentrations using Nanodrop. **c)** We found that substantial amounts of DNA could be amplified from beads incubated with lysed cells, but not from beads incubated with naked DNA or water (negative control). In contrast, DNA amplification was successful from the supernatant separated from the naked DNA mixture. These results suggest that the beads specifically capture genomic DNA, potentially through protein interactions, but not naked DNA.

Supplementary Figure 2

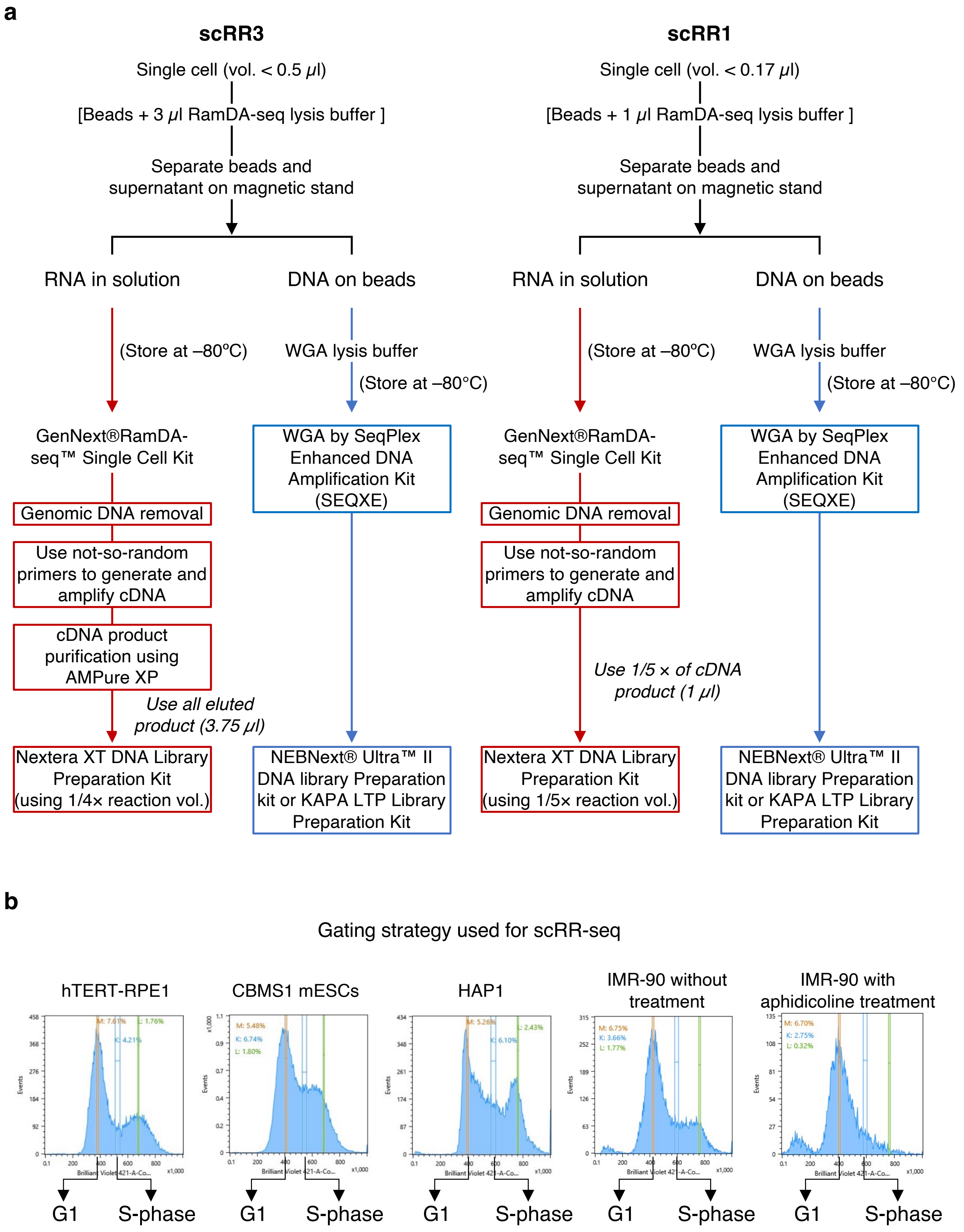

Supplementary Figure 2. Schematic overview of scRR1 and scRR3 workflows.

a) Detail protocols are described in the Methods section in the main text. Single cells were collected into 0.2 ml PCR tubes, followed by lysis using complete RamDA-seq lysis buffer (TOYOBO, RMD-101) containing Dynabeads® MyOne™ Carboxylic Acid (beads). If the initial RamDA-seq lysis buffer volume was 3 μl, we refer to the protocol as scRR3, and if it was 1 μl, we refer to it as scRR1. Since the RamDA-seq lysis buffer is sensitive to excess carryover during cell sorting, which can negatively impact downstream cDNA synthesis, it is recommended that the volume of the cell sample added to the lysis buffer be less than 0.5 μl for scRR3 (as specified in the RamDA-seq manual by TOYOBO). Proportionally, this corresponds to less than 0.17 μl for scRR1. The RNA and DNA fractions were separated using a magnetic stand, with genomic DNA being captured by the beads and RNA remaining in the solution. The RNA fraction was then processed for scRamDA-seq using GenNext®RamDA-seq™ Single Cell Kit (TOYOBO). Both protocols are relatively similar. After removing genomic DNA contamination, cDNA was directly amplified from RNA using not-so-random primers and strand displacement amplification[2]. In the scRR3 protocol, a cDNA purification step using AMPure XP beads is required before library preparation. It is important to completely remove all ethanol during this step, as residual ethanol can result in low library yields. However, in the scRR1 protocol, this purification step can be skipped, allowing direct library preparation. For scRR1, only 1 μl (1/5× of cDNA product) is used for library preparation, as this is optimized for the 1/5× scaled-down Nextera XT DNA Library Preparation Kit. Despite using a small volume, there is no notable drop in data quality or gene detection. As shown in Fig. 1e, scRR1 detects only slightly fewer transcripts and genes than scRR3 (less than 5% difference). This is because the RamDA-seq method amplifies cDNA during reverse transcription using the RT-RamDA approach, which can increase the cDNA amount by more than 10-fold in just 30 minutes[2]. This strong amplification ensures that there is sufficient cDNA material for library preparation. For library preparation, we used 1/4× and 1/5× reaction volumes of Nextera XT DNA Library Preparation Kit for scRR3 and scRR1, respectively. Meanwhile, the genomic DNA captured on the beads was processed in the same manner for both protocols (scRR3 and scRR1). The isolated DNA was subjected to whole-genome amplification (WGA) using SeqPlex Enhanced DNA Amplification Kit (SEQXE), followed by library preparation using NEBNext® Ultra™ II DNA library Preparation kit for Illumina® or KAPA LTP Library Preparation Kit. **b)** scRR-seq gating strategy. Live cells stained with Hoechst 33342 were FACS-sorted. G1 and S phase

populations were defined based on the Brilliant Violet 421 histogram, with the corresponding cells indicated on the plot.

Supplementary Figure 3

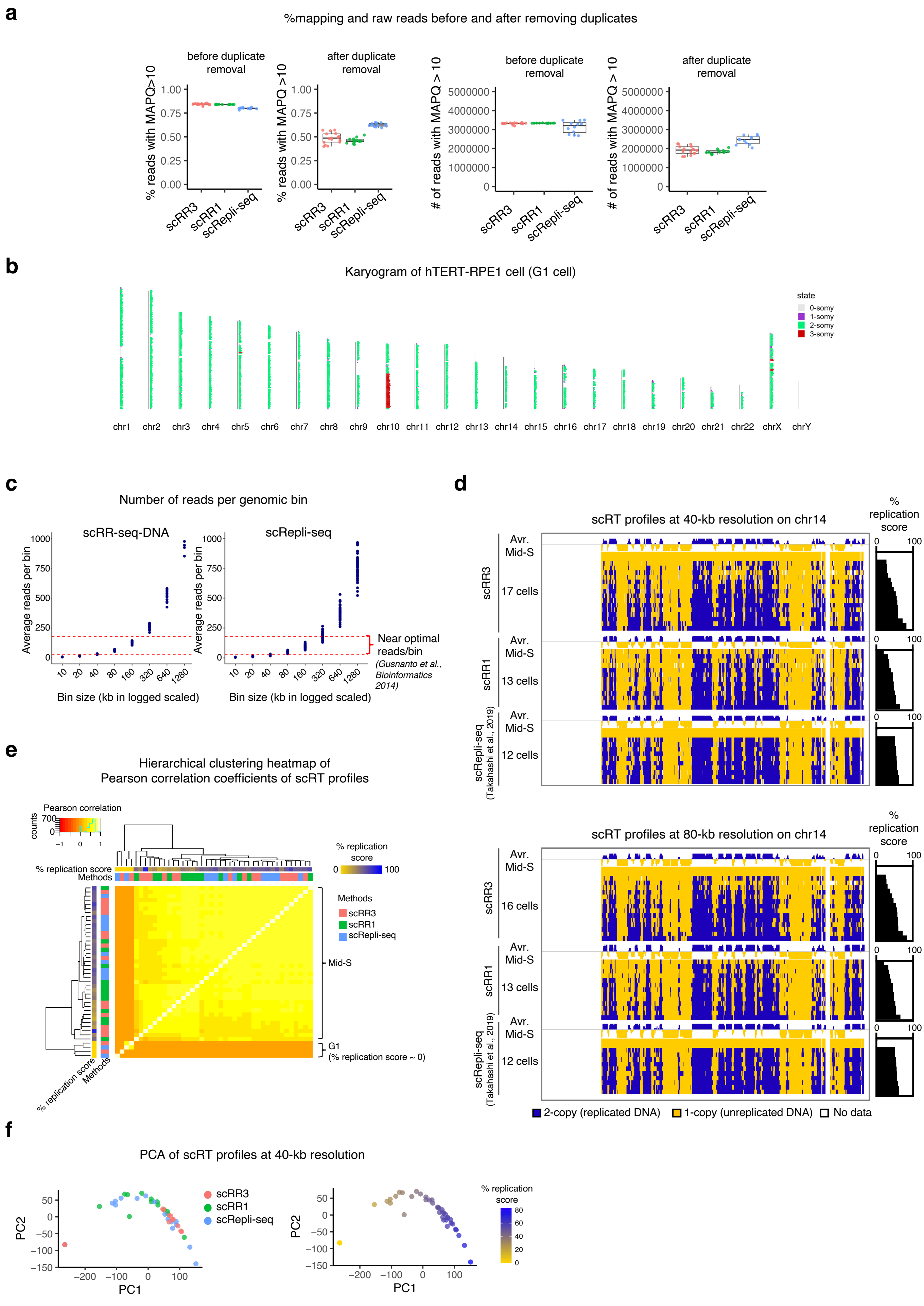

Supplementary Figure 3. Characterization of scRR-seq-DNA.

a) Percentage of reads mapped to hg38 and the corresponding read counts before and after removing duplicates for scRR-seq-DNA (scRR1 and scRR3) and scRepli-seq samples. **b)** Karyogram of a representative G1-phase hTERT-RPE1 cell derived from scRR-seq-DNA. Green and red indicate 2-copy and 3-copy regions, respectively. **c)** Number of reads per genomic bin (10 to 1,280-kb bins). Near-optimal read depths per bin were defined according to Gusnanto et al[3]. **d)** scRT profiles of chromosome 14 (chr14) in hTERT-RPE1 cells at 40-kb (upper panel) and 80-kb (lower panel) resolution. Average RT profiles of mid-S cells (40–70% replication scores) are shown at the top of each method (Avr. Mid-S). One cell is missing in scRR3 at 80-kb resolution due to quality control failure at 80 kb but not 40 kb. scRepli-seq data are from Takahashi et al[4]. **e)** Hierarchical clustering heatmap based on Pearson correlation coefficients of whole genome scRT profiles at 40-kb resolution. While G1-phase cells are clearly distinguishable from mid-S-phase cells, there is no separation between mid-S scRT profiles obtained from different methods, suggesting that scRT profiles are similar across methods. **f)** Principal component analysis (PCA) of whole genome scRT profiles at 40-kb resolution.

Supplementary Figure 4

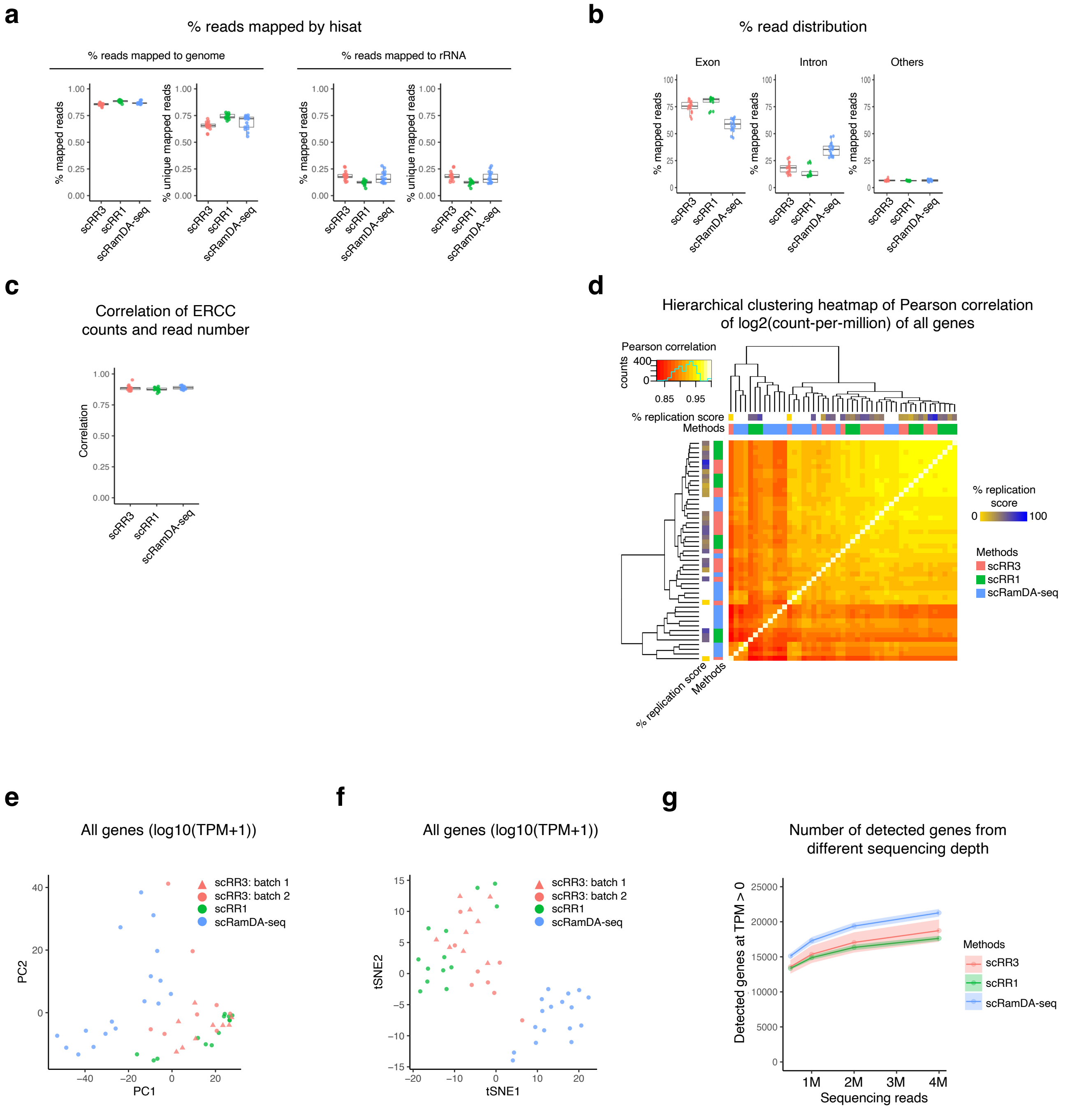

Supplementary Figure 4. scRR-seq-RNA characterization.

a) Percentages of mapped and uniquely mapped reads to hg38 and human ribosomal RNA (rRNA) for scRR-seq-RNA and scRamDA-seq samples. **b)** Percentage of read distribution to exon, intron, and intergenic regions (others). **c)** Strong correlation between ERCC counts and read numbers. **d)** Hierarchical clustering heatmap of Pearson correlation coefficients for gene expression profiles, showing high correlation across samples from all three methods. This

suggests that transcriptome profiles are consistent across methods. **e,f)** Principal component analysis (PCA) (e) and t-distributed stochastic neighbor embedding (tSNE) (f) plots of gene expression profiles. **g)** Detection rates of genes expressed (TPM>0; based on RSEM) in mid-S-phase hTERT-RPE1 cells at varying sequencing read depths. Five cells per method were randomly sampled, and their reads were subsampled to 0.5, 1, 2, and 4 million reads per sample prior to analysis using the ramdaq pipeline. Each line represents a different method. The line and color-shaded areas represent means and SDs, respectively. At TPM>0, the minimum gene detection was 13,344.4 ± 180.7 genes (mean ± SD).

Supplementary Figure 5

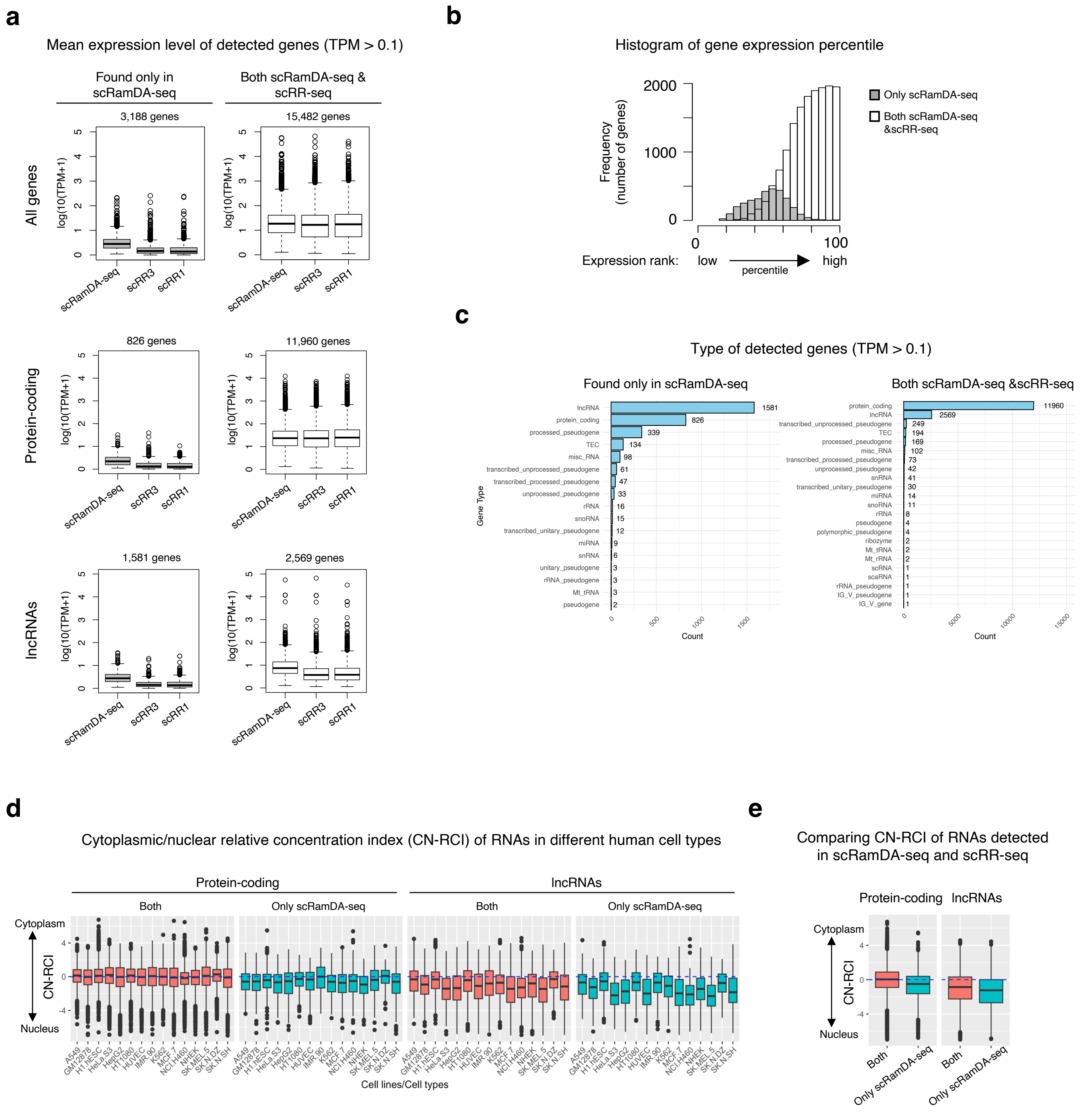

Supplementary Figure 5. Differences between RamDA-seq and scRR-seq-RNA.
a) Expression levels [log10(TPM+1)] of genes detected by only scRamDA-seq or by both scRamDA-seq and scRR-seq. Genes with TPM >0.1 were considered detected. Genes detected only by scRamDA-seq tend to exhibit low expression levels. **b)** Distribution of genes into expression-level percentiles. Genes detected only by scRamDA-seq were enriched in the lower percentiles, indicating a tendency toward low expression levels. **c)** Types of

genes detected only by scRamDA-seq or by both scRamDA-seq and scRR-seq. Genes with TPM >0.1 were considered detected. **d)** Using the list of protein-coding and lncRNA genes from (c), we examined the subcellular localization of their RNAs across various human cell types by utilizing the Cytoplasmic/Nuclear Relative Concentration Index (CN-RCI) from published data[5]. A high CN-RCI indicates cytoplasmic localization, whereas a low CN-RCI indicates nuclear localization. **e)** Based on the results from (d), aggregated CN-RCI values across all cell types were used to generate an overall RNA localization profile for each group, facilitating comparison between groups of genes.

Supplementary Figure 6

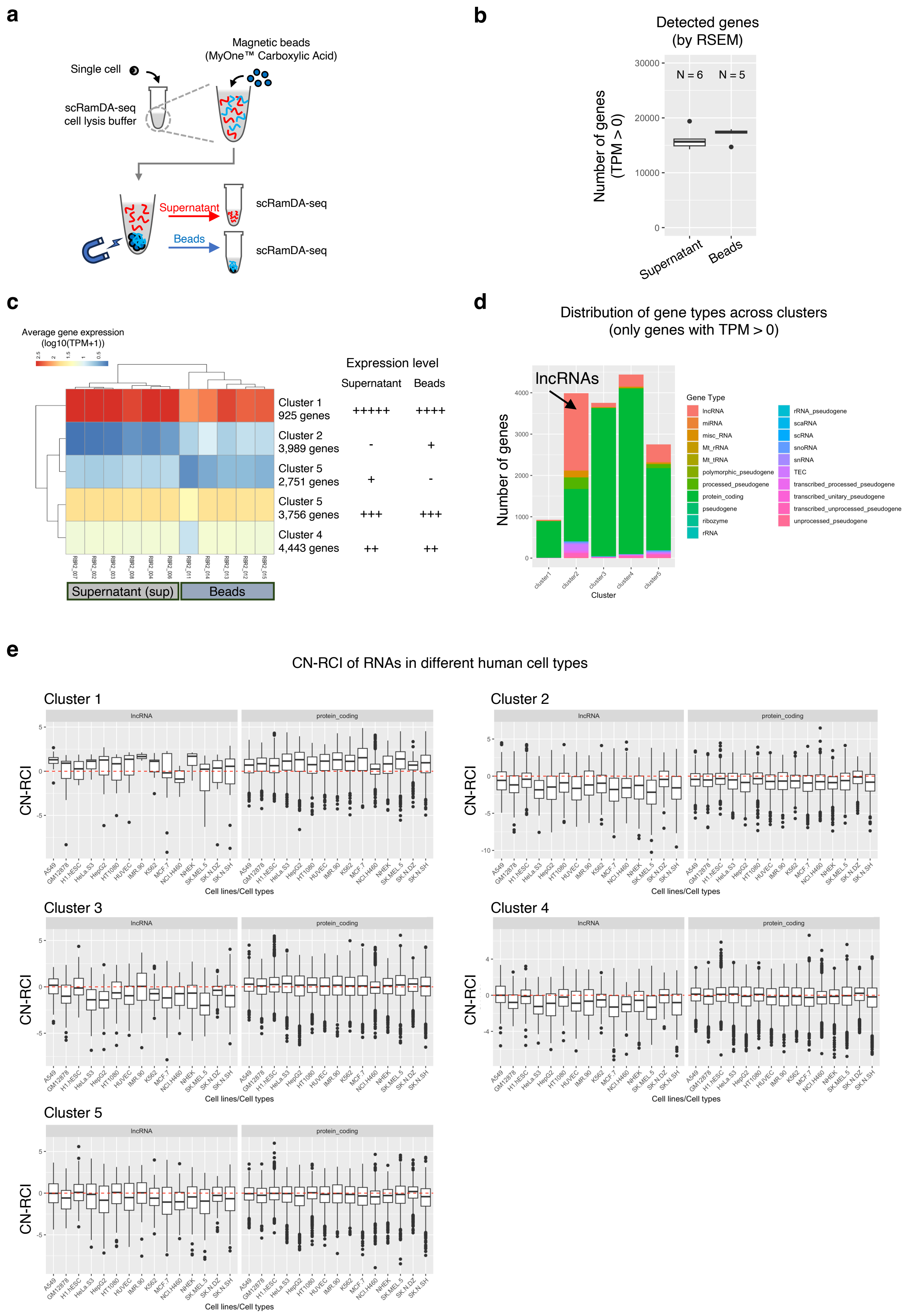

Supplementary Figure 6. RNAs in supernatant and bead-bound fractions.

a) scRamDA-seq was performed using beads or supernatant fractions. **b)** Gene expression quantification by RSEM revealed that fewer genes were detected in the supernatant-derived (cytoplasmic) samples, suggesting that certain RNAs are more enriched in the bead (nuclear) fraction. N indicates sample size. **c)** Clustering analysis revealed that cluster 2 consists of genes with higher expression in the bead fraction than in the supernatant. **d)** Genes in cluster 2 were significantly enriched for lncRNAs, indicating that a subset of lncRNAs preferentially associates with the bead (nuclear) fraction. **e)** CN-RCI of RNAs in different human cell types. Using published RNA localization data in different human cell lines[5], we found that RNAs from genes in cluster 2 tend to be nuclear-localized. This is consistent with the fact that, in scRR-seq-RNA, RNA was collected from the supernatant, which mainly corresponds to the cytoplasmic fraction.

Supplementary Figure 7

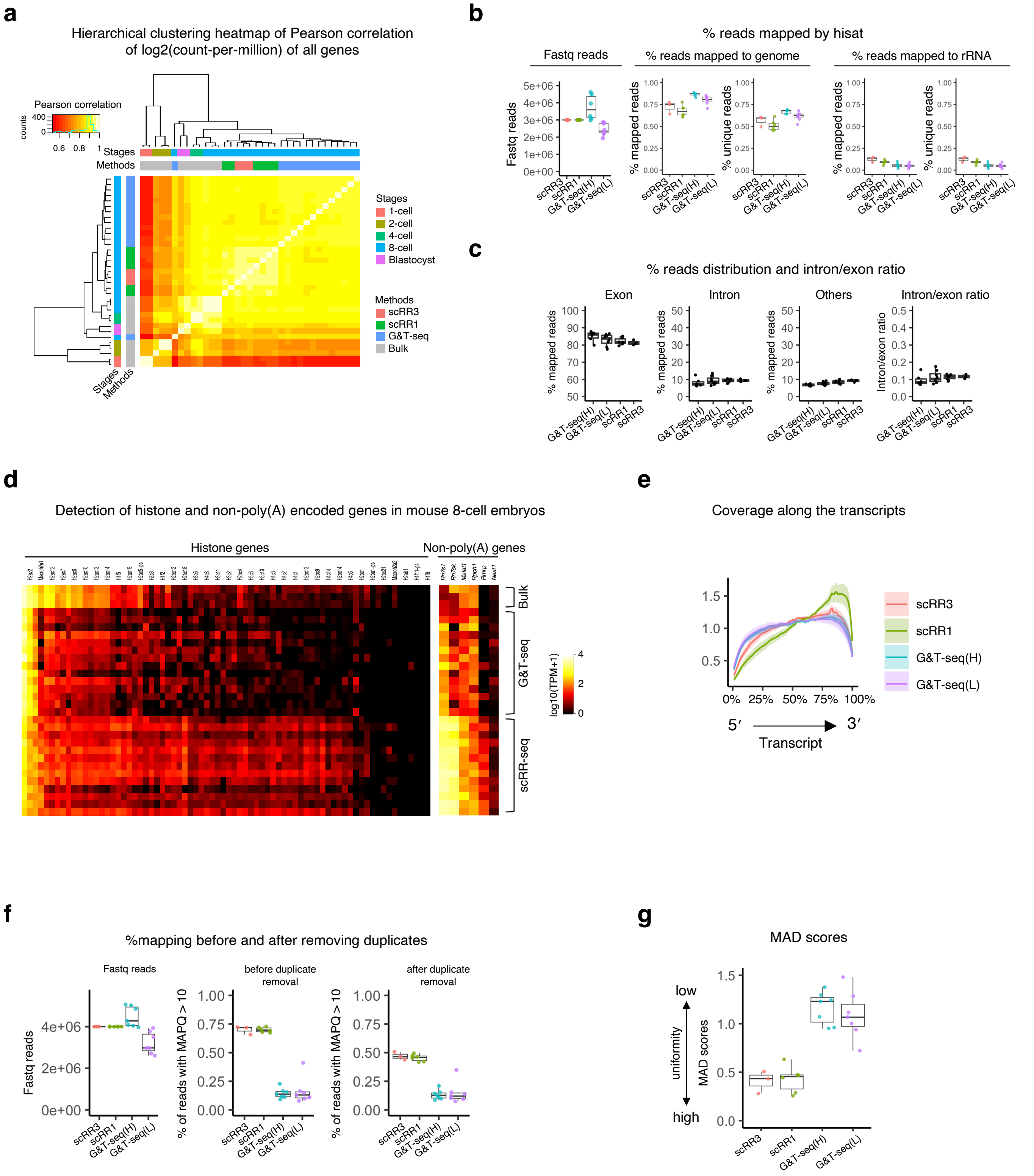

Supplementary Figure 7. Further comparison of scRR-seq and G&T-seq.

a) Hierarchical clustering heatmap of Pearson correlation coefficients of gene expression profiles. The analysis distinguished different stages of mouse preimplantation embryos and

revealed high similarity among 8-cell mouse embryo datasets, despite being generated by different methods. **b)** Fastq read counts, and percentages of reads mapped and uniquely mapped to the mm10 reference genome and mouse rRNA for scRR-seq-RNA and G&T-seq-RNA samples. G&T-seq data[6] were divided into two groups based on initial read counts. G&T-seq(H) includes seven samples with read counts comparable to or higher than those of scRR-seq-DNA, while G&T-seq(L) includes seven samples with lower read counts than scRR-seq-DNA. scRR3 and scRR1 sample sizes are three and six, respectively. **c)** Percentage read distribution to exon, intron, intergenic regions, and the intron/exon ratio. A slight shift in the intron/exon ratio was observed in scRR-seq. **d)** Expression levels of histone and known non-poly(A) encoded genes in 8-cell mouse embryos across different methods. scRR-seq captured these genes more effectively than both bulk RNA-seq and G&T-seq methods. **e)** Mean read coverage along transcripts. **f)** Percentage of reads mapped to mm10 and corresponding read counts before and after removing duplicates for scRR-seq-DNA (scRR1 and scRR3) and G&T-seq-DNA samples. **g)** MAD scores of scRR-seq-DNA and G&T-seq-DNA samples.

Supplementary Figure 8

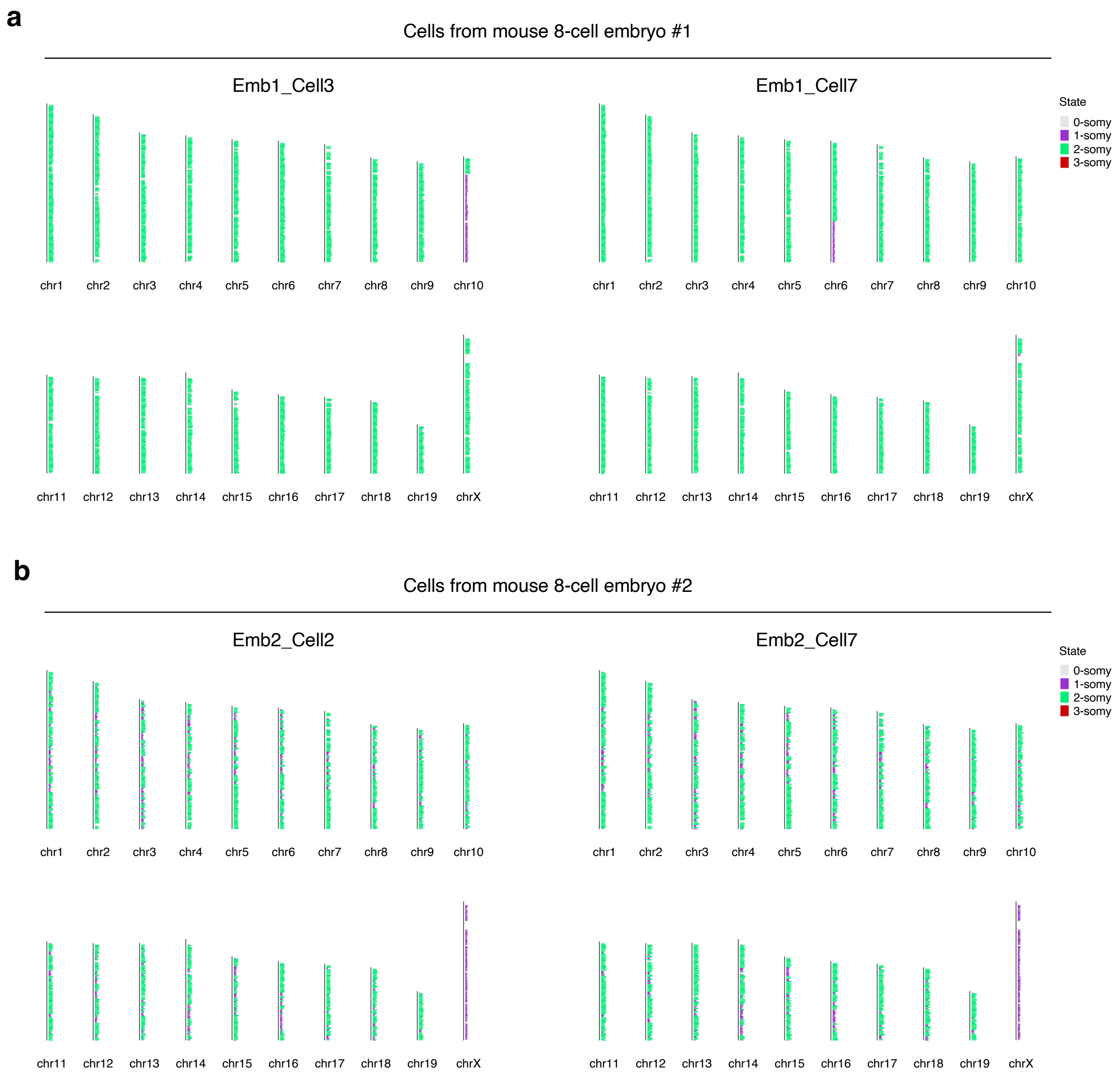

Supplementary Figure 8. Karyograms of cells isolated from 8-cell mouse embryos. Single cells isolated from 8-cell mouse embryos were subjected to scRR-seq-DNA. After mapping to the mm10 reference genome and removing low-quality reads, we applied 6-state Hidden Markov Model (HMM) calling using AneuFinder with 500-kb bins. The resulting data were visualized as karyograms, with colors indicating HMM-derived copy number states. Representative karyograms from four cells across two embryos are shown. **a)** Representative cells from embryo #1 displayed uniform two-copy profiles across the genome (except for regions with chromosomal abnormalities). These cells were classified as being in

either G1 or G2/M phase. **b)** Representative cells from embryo #2 exhibited alternating one- and two-copy number states across the genome, consistent with S-phase DNA replication.

Supplementary Figure 9

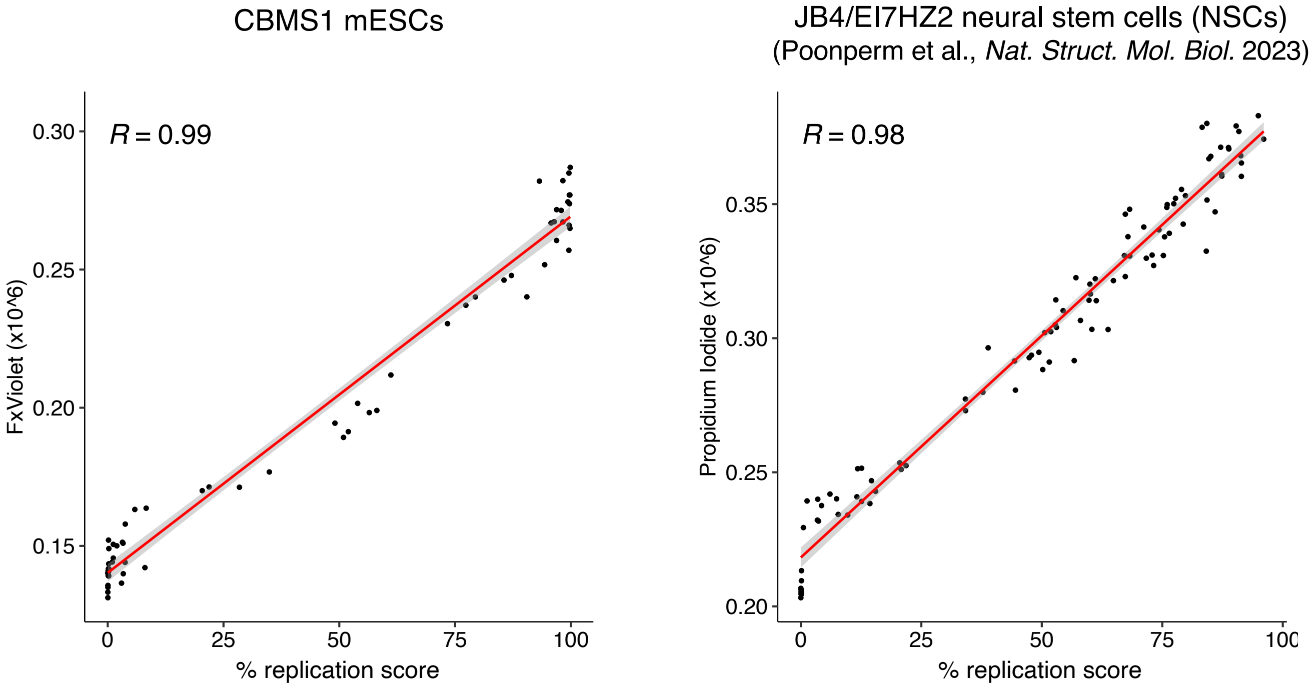

Supplementary Figure 9. Strong correlation between DNA content and percentage replication score.

Single cells from CBMS1 mESCs[4] (left) and JB4 mouse neural stem cells[7] (NSCs) were fixed with 75% ethanol and stained with the DNA-binding dyes FxViolet and Propidium Iodide, respectively. The stained cells were sorted individually using a SONY SH800 sorter with index function, which recorded their fluorescent intensity. The sorted cells were then subjected to WGA and analyzed for their percentage replication score using the scRepli-seq protocol. We plotted the fluorescent intensity against the percentage replication score for each individual cell and found a high correlation ($R > 0.98$). This suggests that the percentage replication score derived from scRepli-seq accurately reflects the cell-cycle timepoint of each cell.

Supplementary Figure 10

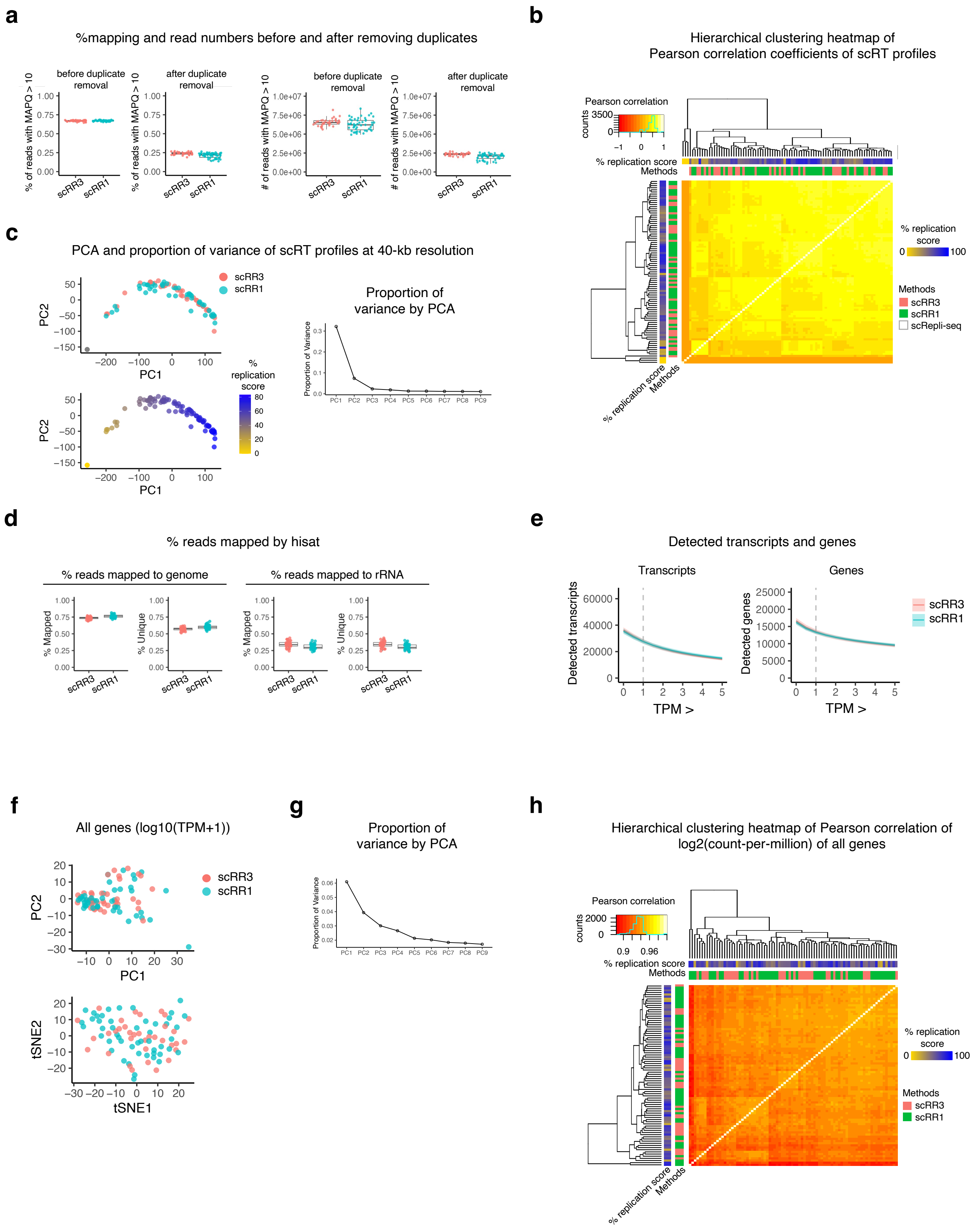

Supplementary Figure 10. scRR-seq analysis of CBMS1 mESCs.

We performed two scRR-seq methods, scRR1 and scRR3, on CBMS1 mESCs. **a)** Percentage of reads mapped to the mm10 reference genome and corresponding read counts before and after removing duplicates for scRR-seq-DNA (scRR1 and scRR3) in CBMS1 mESCs. **b)** Hierarchical clustering heatmap of Pearson correlation coefficients of whole-genome scRT profiles at 40-kb resolution, showing high similarity between scRR1 and scRR3. **c)** PCA and the proportion of variance explained by each principal component of CBMS1 mESC scRT profiles (whole genome) at 40-kb resolution. **d)** Percentages of mapped and uniquely mapped reads to the mm10 mouse genome and mouse rRNA for scRR1 and scRR3 samples. **e)** Detection rates of transcripts and genes expressed in CBMS1 mESCs at different expression level thresholds in each method. Lines and color-shaded areas represent means and SDs, respectively. scRR1 and scRR3 showed similar transcript and gene detection levels. **f)** PCA and tSNE plots of gene expression profiles for CBMS1 mESCs. **g)** Proportion of variance by PCA shown in (f). **h)** Hierarchical clustering heatmap of Pearson correlation coefficients of gene expression profiles, demonstrating high similarity across samples despite being derived from different scRR-seq protocols.

Supplementary Figure 11

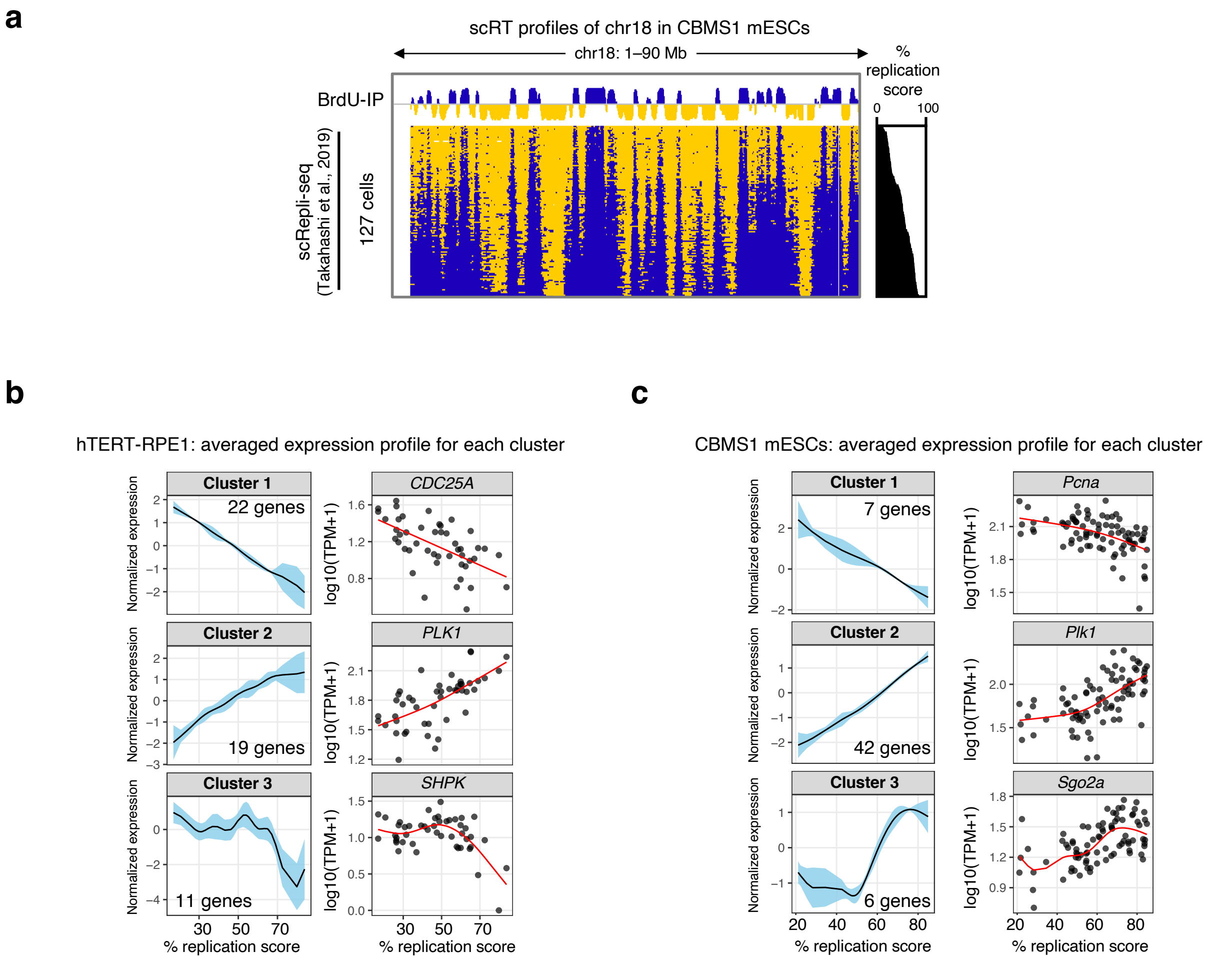

Supplementary Figure 11. Cell-cycle and S-phase progression markers in hTERT-RPE1 cells and CBMS1 mESCs.

a) Whole-S scRT profiles of CBMS1 mESCs derived from scRepli-seq[13]. Chromosome 18 (chr18) is shown. An RT profile from BrdU-IP Repli-seq is shown at the top. **b)** and **c)** Identification of genes that show dynamic changes during S-phase progression in hTERT-RPE1 cells and CBMS1 mESCs using scRR-seq. The left panels show the average expression profiles of each gene cluster. The number of genes in each cluster is indicated. The x-axis represents the percentage replication score. The color-shaded areas represent SDs. The right panels show the expression profiles of representative genes from each cluster, with the red lines representing a fitted generalized additive model (GAM). Dots show single-cell gene expression levels [log10(TPM+1)]. The x-axis represents the percentage replication score.

Supplementary Figure 12

a

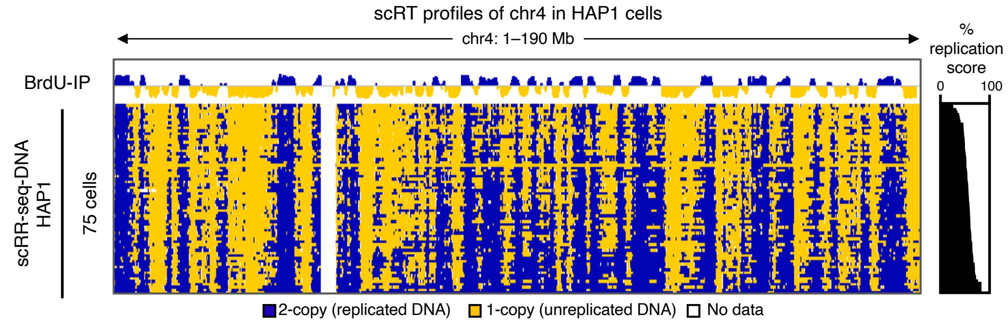

b

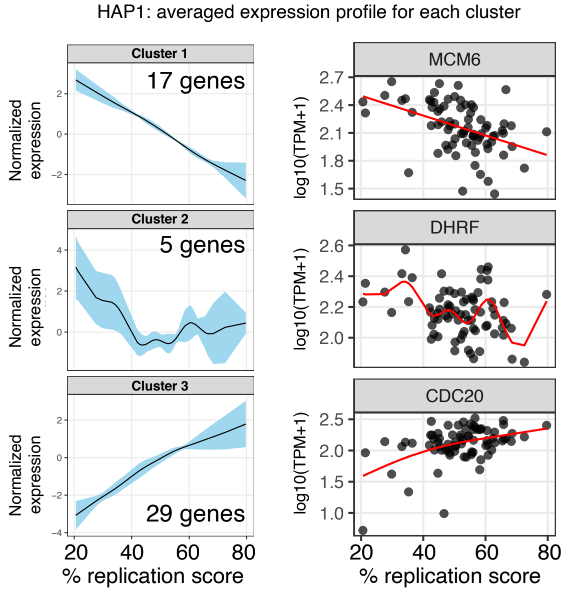

c

Novel S-phase progression markers identified in HAP1

gene_id	cluster	Length	gene_name
ENSG00000010539.12	1	7498	ZNF200
ENSG00000015285.11	2	2361	WAS
ENSG00000059377.18	1	6271	TBXAS1
ENSG00000073578.17	3	8765	SDHA
ENSG00000076003.5	1	4135	MCM6
ENSG00000100297.16	1	7268	MCM5
ENSG00000104738.18	1	6973	MCM4
ENSG00000110011.13	3	2807	DNAJC4
ENSG00000112118.20	1	3645	MCM3
ENSG00000115194.11	3	5766	SLC30A3
ENSG00000115361.8	1	5152	ACADL
ENSG00000116062.17	1	10955	MSH6
ENSG00000116161.18	1	4611	CACYBP
ENSG00000117399.14	3	2012	CDC20
ENSG00000130584.12	3	7086	ZBTB46
ENSG00000140451.13	3	4656	PIF1
ENSG00000147144.13	3	5318	CCDC120
ENSG00000155099.8	3	2868	PIP4P2
ENSG00000163412.13	2	10808	EIF4E3
ENSG00000163703.20	3	9266	CRELD1
ENSG00000165985.10	2	2376	C1QL3
ENSG00000166197.17	1	3970	NOLC1
ENSG00000168056.17	3	10675	LTBP3
ENSG00000169992.10	3	5717	NLGN2
ENSG00000170035.16	3	2974	UBE2E3
ENSG00000170540.15	3	3558	ARL6IP1
ENSG00000171604.12	3	3795	CXXC5
ENSG00000172819.17	3	5127	RARG
ENSG00000173801.17	3	4942	JUP
ENSG00000174606.14	1	7317	ANGEL2
ENSG00000177685.17	3	4224	CRACR2B
ENSG00000178878.13	3	7191	APOLD1
ENSG00000182481.10	3	7365	KPNA2
ENSG00000183044.12	3	9713	ABAT
ENSG00000183963.19	3	10242	SMTN
ENSG00000186676.3	3	1299	EEF1GP1
ENSG00000196712.18	3	27130	NF1
ENSG00000198081.11	1	4144	ZBTB14
ENSG00000198182.13	1	4703	ZNF607
ENSG00000198440.9	1	8060	ZNF583
ENSG00000198938.2	3	784	MT-CO3
ENSG00000198952.8	1	5304	SMG5
ENSG00000204305.15	3	2075	AGER
ENSG00000228716.7	2	4411	DHFR
ENSG00000241404.7	3	2609	EGFL8
ENSG00000248550.4	3	3505	OTX2-AS1
ENSG00000272954.1	3	430	KB-1440D3.13
ENSG00000273748.1	2	372	ENSG00000273748
ENSG00000276043.5	1	5145	UHRF1
ENSG00000278784.1	3	1200	RP11-468E2.11
ENSG00000280332.1	1	1999	CTD-2013N17.6

d

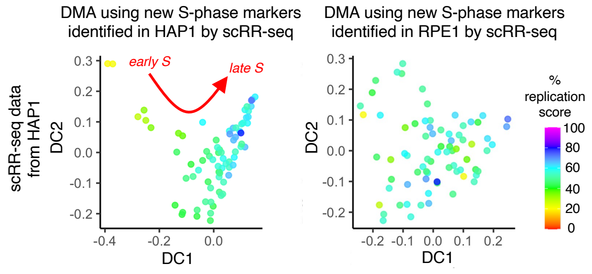

e

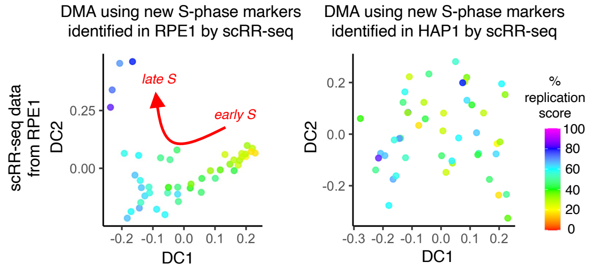

f

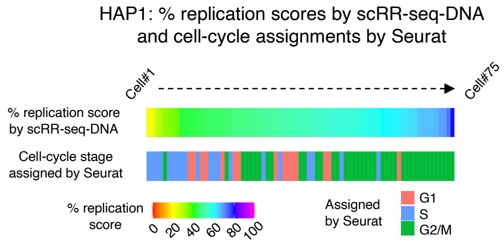

Supplementary Figure 12. S-phase gene expression dynamics in HAP1 cells.

We also performed scRR-seq in human haploid HAP1 cells and examined S-phase gene expression dynamics and identifying S-phase progression markers. **a)** Whole-S scRT profiles of human haploid HAP1 cells derived from scRR-seq-DNA. Chromosome 4 (chr4) is shown. The population-based BrdU-IP Repli-seq RT profile is shown at the top for comparison. **b)** scRR-seq-RNA data of these cells were analyzed to identify novel S-phase progression marker candidates, using the same approach as in hTERT-RPE1 cells (Fig. 3 and Supplementary Fig. 11). **c)** List of novel S-phase progression markers identified in HAP1 cells by scRR-seq. **d,e)** Utilizing these newly identified genes for diffusion map analysis (DMA), we observed a clear trajectory of S-phase progression in HAP1 cells. However, when using S-phase progression markers identified in hTERT-RPE1 cells, we were unable to reveal S-phase progression trajectory in HAP1 and vice versa, suggesting the cell-type specificity of markers. Each dot represents individual cell, which is color-coded by its % replication score. DMA of hTERT-RPE1 and HAP1 data using newly identified S-phase progression markers by scRR-seq as indicated. **f)** Comparison between scRR-seq-DNA's percentage replication scores and Seurat's cell-cycle phase assignments using its default cell-cycle markers in HAP1 cells. Similar to what was observed in hTERT-RPE1 (Fig. 3e), despite HAP1 cells being in S-phase based on the percentage replication scores, Seurat assigned them to G1, S, and G2/M phases. While the S and G2/M phase assignments were relatively reasonable and marked cells with low and high percentage replication scores, respectively, some cells were assigned as G1.

Supplementary Figure 13

a

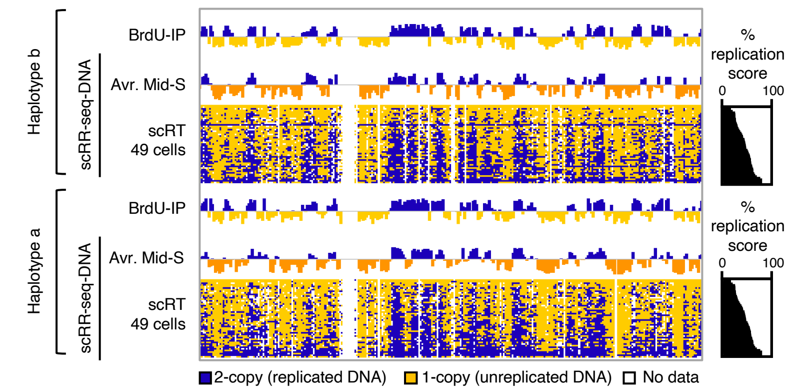

b

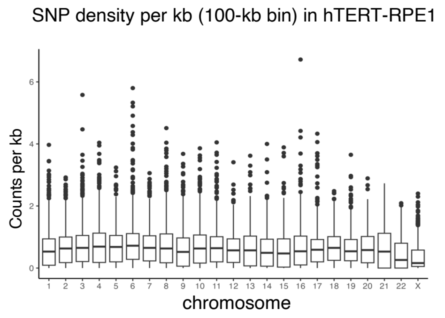

c

Informative genes in hTERT-RPE1 (excluded chr10)

Category	Number of informative genes
Biallelic	1808
Monoallelic	38 (14 X-linked genes)
Biased to haplotype-a	15
Biased to haplotype-b	17

d

Informative genes in CBMS1 mESCs (excluded chr8 and chrX)

Category	Number of informative genes
Biallelic	5512
Monoallelic	171
Biased to CBA	134
Biased to MSM	155

e

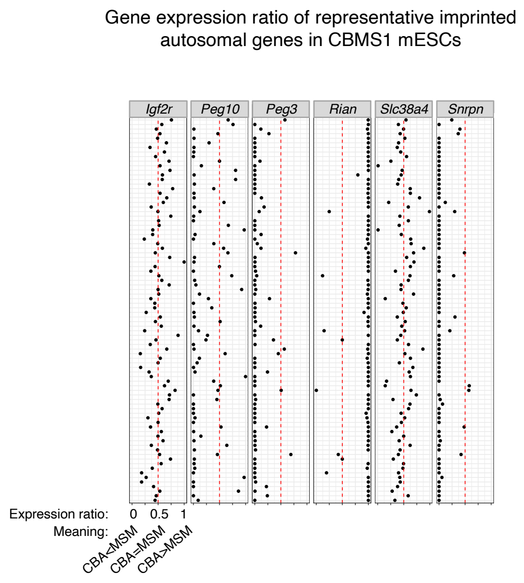

f

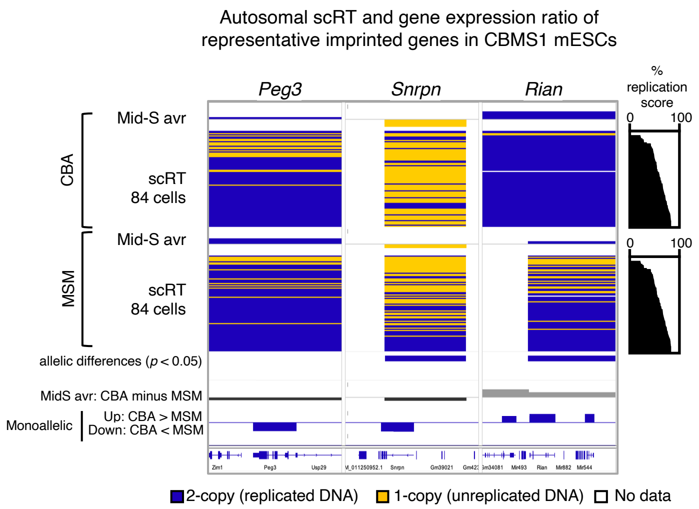

Supplementary Figure 13. Haplotype-specific analysis in hTERT-RPE1 cells and CBMS1 mESCs

a) Haplotype-specific scRT profiles of a representative autosome (chromosome 12 (chr12)) in hTERT-RPE1 cells, derived from scRR-seq-DNA at 400-kb resolution. For comparison,

haplotype-specific BrdU-IP RT profiles are also shown. Average mid-S RT profiles are based on cells with 40–70% replication scores (Avr. Mid-S). **b)** SNP density on chrX is lower than that on autosomes. SNP density was calculated for every 100-kb bin. **c)** The number of haplotype-specific informative genes in hTERT-RPE1 cells for each gene category. **d)** The number of allele-specific informative genes in CBMS1 mESCs for each gene category. **e)** Allele-specific gene expression ratios of representative known imprinted autosomal genes in CBMS1 mESCs. Each row displays the allele-specific gene expression ratio in each cell (black dot). **f)** scRT profiles and allele-specific gene expression ratios of representative imprinted genes in CBMS1 mESCs.

Supplementary Figure 14

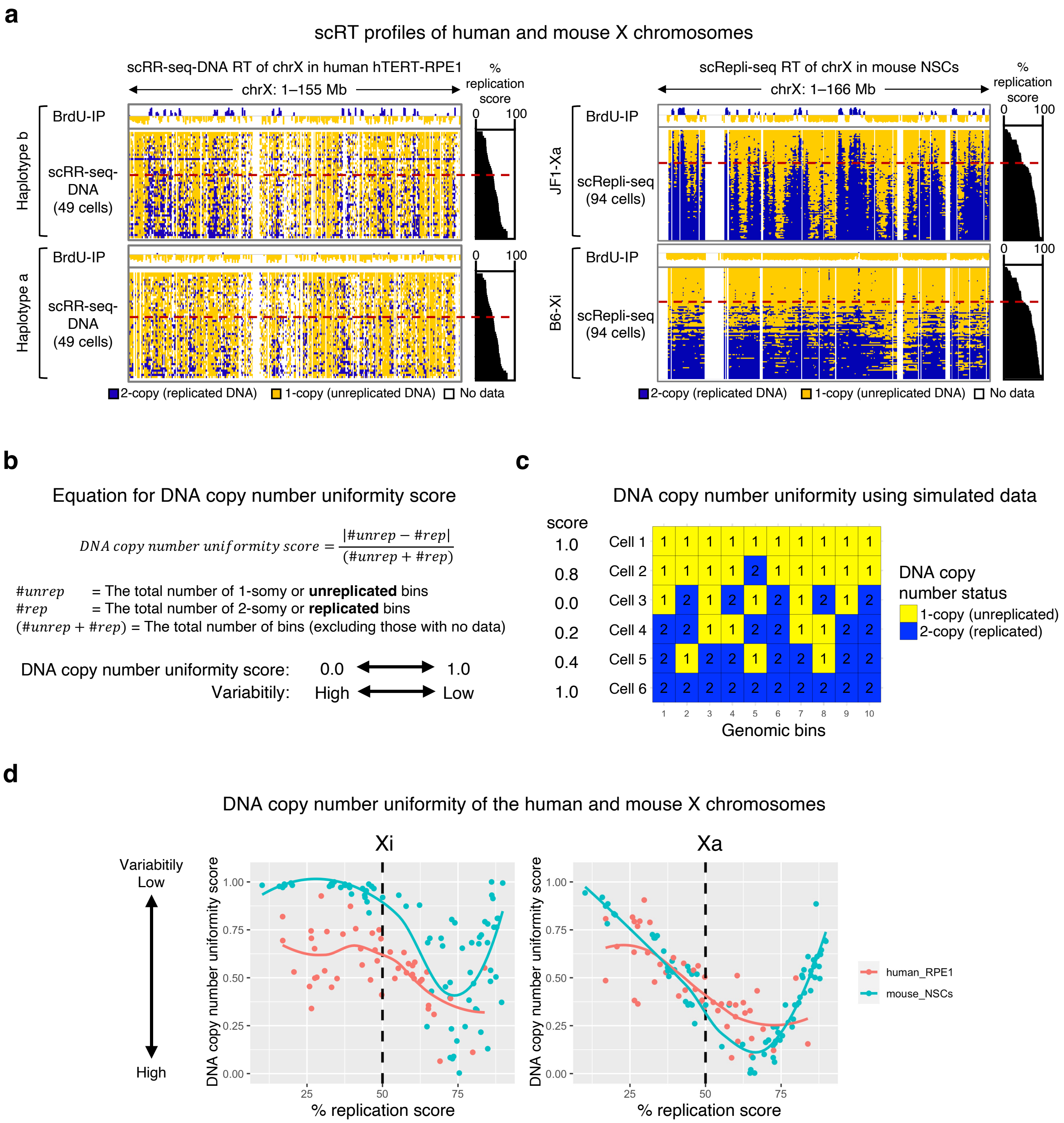

Supplementary Figure 14. RT of the X chromosome in female human and mouse cells. a) scRT profiles of the X chromosome in human RPE1 cells (left) and mouse JB4/EI7HZ2 NSCs[7] (right). Red dashed lines indicate the 50% replication score (mid-S). **b)** An equation used to calculate DNA copy number uniformity score. The score is calculated by dividing the absolute difference between the number of 1-copy (unreplicated) and 2-copy (replicated) bins

by the total number of counted bins, excluding bins with no data. The score ranges from 0.0 to 1.0, where 1.0 indicates complete uniformity (all bins are either entirely 1-copy or entirely 2-copy), and 0.0 reflects maximum variability (an equal number of 1-copy and 2-copy bins). **c)** Example of DNA copy number uniformity score usage. Suppose we have 6 cells and 10 genomic bins, with their DNA copy number status illustrated in the figure. Bins with 1-copy (unreplicated) and 2-copy (replicated) states are shown in yellow and blue, respectively, and are numerically encoded as 1 and 2. Cells exhibiting consistent copy number across all bins, such as cells 1 and 6, which are entirely 1-copy or entirely 2-copy, show a uniformity score of 1.0, reflecting complete uniformity in DNA replication status across the genomic region. In contrast, cells with a mix of 1- and 2-copy bins have lower uniformity scores, indicating variability of replication states within the cell. **d)** DNA copy number uniformity of the X chromosomes. Each dot represents DNA copy number uniformity score of the Xi (left) and Xa (right) in individual human RPE1 cells or mouse NSCs[7]. Lines show loess smoothing of the data for human and mouse cells. As expected, we observed that the mouse Xi shows high uniformity in early- and late-S phases, but lower uniformity in mid-S. In contrast, the human Xi showed relatively lower DNA copy number uniformity score across all S-phase stages, compared to the mouse Xi. This suggests that replication on the human Xi is less uniform than on the mouse Xi. We also performed the same analysis on Xa in both species. In human and mouse cells, the Xa exhibited relatively similar DNA copy number uniformity across S-phase stages. These results indicates that the differences observed are specific to Xi. While these findings support the conclusion that the human Xi exhibits less uniform late replication compared to mouse Xi, it is important to consider that technical factors such as SNP density or mappability could also contribute to these observations.

Supplementary Figure 15

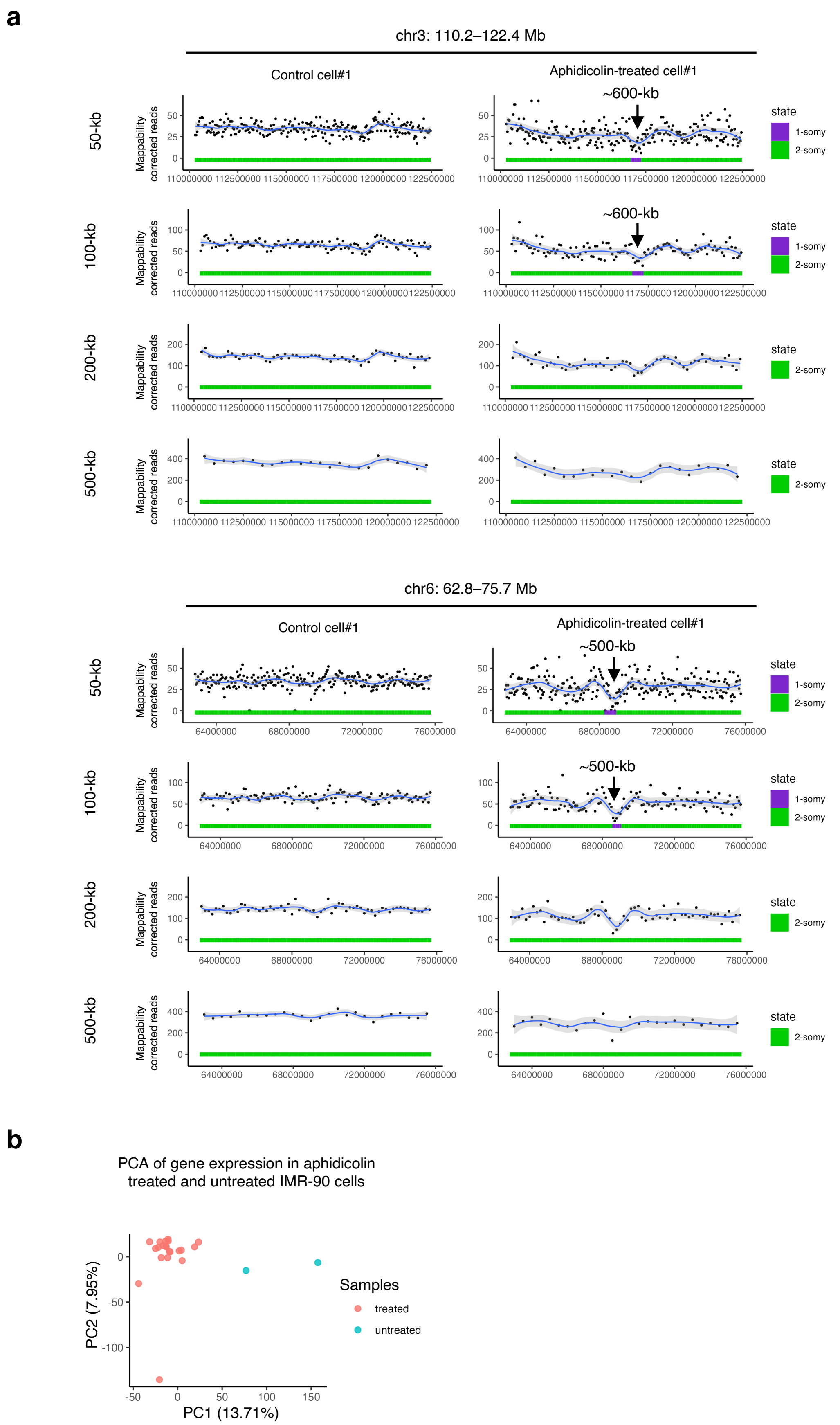

Supplementary Figure 15. Copy-number variations (CNVs) in aphidicolin-treated IMR-90 cells.

a) Mappability-corrected read counts for each indicated bin size are shown. The corresponding CNVs are shown at the bottom of each plot. CNV positions are shown by arrows, and their sizes are indicated. Each blue line represents loess smoothing (span = 0.2 for 50-kb, 100-kb, and 200-kb; span = 0.4 for 500-kb bins). Shaded area represents SDs. **b)** PCA of gene expression profiles in aphidicolin-treated and untreated IMR-90 cells. Only genes with TPM>1 in more than 50% of IMR-90's scRR-seq-RNA samples were used for the analysis.

Supplementary Notes

Supplementary Note 1: When to use scRR1 vs. scRR3

When using cells sorted by FACS, both scRR1 and scRR3 yielded comparable results (see Fig. 1 and Supplementary Fig. 3 for hTERT-RPE1; Supplementary Fig. 10 for CBMS1 mESCs). However, when cells were manually isolated from early mouse embryos, significant differences were observed between the scRR1 and scRR3 methods, with the latter generating higher-quality RNA-seq data. scRR1 exhibited a 3′-end bias in transcript coverage (Supplementary Fig. 7e) and detected fewer transcripts than scRR3 (Fig. 2b). This discrepancy may result from solution carryover during manual cell handling, which can interfere with downstream cDNA synthesis (see details in Supplementary Fig. 2). Thus, the 1 μl volume of RamDA lysis buffer used in scRR1 may be a suboptimal condition for manually isolated samples. In contrast, scRR-seq-DNA data remained consistent regardless of the protocol or sample handling method.

Given that scRR1 uses smaller reagent volumes and requires fewer handling steps compared to scRR3, it is more suitable for large-scale experiments without carryover volume issues. In contrast, for applications requiring high-quality RNA-seq data, particularly when manual cell isolation is involved, scRR3 may be a better choice. A detailed side-by-side comparison is summarized in the Supplementary Table 1 and illustrated in Supplementary Fig. 2a.

Supplementary Note 2: Discussion of RT of the X chromosomes in hTERT-RPE1 cells

Despite the variability observed in the scRT profiles, we found that haplotype-a of the X chromosome (chrX) replicates later, whereas haplotype-b replicates earlier (Fig. 4a). Because this distinction was not entirely clear from the scRT profiles alone, we validated the trend by generating average mid-S RT profiles for each haplotype (Fig. 4a).

The average mid-S RT profile of haplotype-b of chrX (obtained from cells with 40–70% replication scores) showed early and late RT domains similar to those observed in haplotype-specific BrdU-IP RT profiles. However, the early RT peaks of haplotype-b were relatively shallow (Fig. 4a), unlike the autosomes, where average mid-S RT profiles closely matched the BrdU-IP data (Supplementary Fig. 13a). This suggests that the whole human active X chromosome (Xa) replicates later than the autosomes. Furthermore, the scRT profiles and average mid-S profiles of haplotype-b of chrX (Xa) revealed that the p-arm (left arm) replicates earlier than the rest of the chromosome (Fig. 4a), implying a more euchromatic state of the p-arm on the Xa. Previous studies have shown that human escapees (X-linked genes that remain active even on the Xi) are enriched on the p-arm of the X chromosome[7,8]. These findings suggest that intrinsic chromatin status strongly influences replication dynamics and gene expression on the X chromosome. Notably, neither the overall RT delay of the human Xa nor the earlier replication of its p-arm was evident from population-based haplotype-specific BrdU-IP RT data.

Haplotype-a of chrX, which most likely corresponds to the inactive X chromosome (Xi), exhibited distinct replication behavior. Unlike the mouse Xi in neural stem cells, which displays rapid and uniform late replication[7], the human Xi in hTERT-RPE1 cells did not (Supplementary Fig. 14a). This observation led us to hypothesize that the human Xi exhibits less uniform late replication than the mouse Xi.

To test this, we developed a simple metric, the DNA copy number uniformity score, to quantify the consistency of replication status across bins within a chromosome in individual cells (Supplementary Fig. 14b,c). The logic is as follows: chromosomes that replicate uniformly at a given time point should exhibit similar replication status across most bins, resulting in a higher score. For example, in early S-phase (most regions are unreplicated) and late S-phase (most regions are fully replicated), uniformity is high. In contrast, during mid S-

phase, when replication is already completed in some bins/regions while others remain unreplicated, uniformity is reduced.

Applying this analysis, we found that the mouse Xi followed the expected pattern: high and stable uniformity during early to mid S phase (up to ~60% replication score), followed by a sharp drop to lower uniformity in mid to late-S (~60–80% replication score), before quickly recovering to high uniformity in late-S phase. By contrast, the human Xi exhibited persistently lower copy number uniformity scores across all stages of S-phase, suggesting that replication on the human Xi is more variable (Supplementary Fig. 14d). We also performed the same analysis on the Xa in both species. In both human and mouse cells, the Xa exhibited somewhat similar DNA copy number uniformity patterns: higher uniformity during early and late S phases, and lower uniformity in mid S, although the pattern was less clear in human cells (Supplementary Fig. 14d).

While this may reflect true biological differences, technical factors such as SNP density or mappability could also contribute to these observations. Nonetheless, these results again underscore the limitations of population-based haplotype-specific BrdU-IP data, which could not clarify these differences. These results highlight the advantage of using scRepli-seq data.

Supplementary Table

Supplementary Table 1. Comparison of scRR1 and scRR3

Feature	scRR1	scRR3
Initial lysis buffer volume	1 µl	3 µl
Workflow complexity	Simple (no cDNA purification step required)	More steps (includes cDNA purification)
Hands-on time per library preparation	2–4 days	2–4 days
Cost per sample (DNA and RNA-seq)	Low (~$60/sample)	High (~$100/sample)
Compatibility with FACS	High	High
Compatibility with manual cell isolation	Low (requires the carryover volume to be <0.17 µl)	High (requires the carryover volume to be <0.5 µl)
Best suited for	Medium- to large-scale studies using FACS	Limited samples and manually isolated samples
Example use case	Cell lines, automation-friendly workflows	Embryo dissection, rare or precious samples
DNA detection sensitivity	High	High
RNA detection sensitivity	Slightly low (~3.5% lower than scRR3 for FACS samples; ~10% lower for manually isolated samples)	High

Supplementary References

1. Evans, E. E. *et al.* High-permeability functionalized silicone magnetic microspheres with low autofluorescence for biomedical applications. *Mater. Sci. Eng. C. Mater. Biol. Appl.* **62**, 860–869 (2016).
2. Hayashi, T. *et al.* Single-cell full-length total RNA sequencing uncovers dynamics of recursive splicing and enhancer RNAs. *Nat. Commun.* **9**, 619 (2018).
3. Gusnanto, A. *et al.* Estimating optimal window size for analysis of low-coverage next-generation sequence data. *Bioinformatics* **30**, 1823–1829 (2014).
4. Takahashi, S. *et al.* Genome-wide stability of the DNA replication program in single mammalian cells. *Nat. Genet.* **51**, 529–540 (2019).
5. Mas-Ponte, D. *et al.* LncATLAS database for subcellular localization of long noncoding RNAs. *RNA* **23**, 1080–1087 (2017).
6. Macaulay, I. C. *et al.* G&T-seq: Parallel sequencing of single-cell genomes and transcriptomes. *Nat. Methods* **12**, 519–522 (2015).
7. Poonperm, R. *et al.* Replication dynamics identifies the folding principles of the inactive X chromosome. *Nat. Struct. Mol. Biol.* **30**, 1224–1237 (2023).
8. Tukiainen, T. *et al.* Landscape of X chromosome inactivation across human tissues. *Nature* **550**, 244–248 (2017).
