# Supplementary material for: scRepli-RamDA-seq: a multi-omics technology enabling the analysis of gene expression dynamics during S-phase
Source: Nat Commun. 2025 Dec 15;16:10902. doi: 10.1038/s41467-025-64688-1 (PMC12705686; doi:10.1038/s41467-025-64688-1)
Supplement: Supplementary file 2 — Description of Additional Supplementary Files [file 41467_2025_64688_MOESM2_ESM.pdf]

Description of Additional Supplementary Files

File Name: Supplementary Data 1
Description: Sample list

File Name: Supplementary Data 2
Description: Newly identified S-phase progression markers in hTERT-RPE1 cells and CBMS1 mESCs

File Name: Supplementary Data 3
Description: Informative genes from haplotype-specific analysis in hTERT-RPE1 cells and allele-specific analysis of CBMS1 mESCs

File Name: Supplementary Data 4
Description: DNA replication status and gene expression values (log10(mapped reads+1)) of genes exhibiting 1-copy or 2-copy states during mid-S phase in HAP1 cells

File Name: Supplementary Data 5
Description: Cost breakdown of scRR-seq
