# Supplementary material for: scRepli-RamDA-seq: a multi-omics technology enabling the analysis of gene expression dynamics during S-phase
Source: Nat Commun. 2025 Dec 15;16:10902. doi: 10.1038/s41467-025-64688-1 (PMC12705686; doi:10.1038/s41467-025-64688-1)
Supplement: Supplementary file 8 — Reporting Summary [file 41467_2025_64688_MOESM8_ESM.pdf]

nature portfolio

Corresponding author(s): Shin-ichiro Takebayashi

Last updated by author(s): Sep 17, 2025

Reporting Summary

Nature Portfolio wishes to improve the reproducibility of the work that we publish. This form provides structure for consistency and transparency in reporting. For further information on Nature Portfolio policies, see our Editorial Policies and the Editorial Policy Checklist.

Statistics

For all statistical analyses, confirm that the following items are present in the figure legend, table legend, main text, or Methods section.

n/a	Confirmed	
☐	☒	The exact sample size (*n*) for each experimental group/condition, given as a discrete number and unit of measurement
☐	☒	A statement on whether measurements were taken from distinct samples or whether the same sample was measured repeatedly
☐	☒	The statistical test(s) used AND whether they are one- or two-sided<br>*Only common tests should be described solely by name; describe more complex techniques in the Methods section.*
☒	☐	A description of all covariates tested
☒	☐	A description of any assumptions or corrections, such as tests of normality and adjustment for multiple comparisons
☐	☒	A full description of the statistical parameters including central tendency (e.g. means) or other basic estimates (e.g. regression coefficient) AND variation (e.g. standard deviation) or associated estimates of uncertainty (e.g. confidence intervals)
☐	☒	For null hypothesis testing, the test statistic (e.g. *F*, *t*, *r*) with confidence intervals, effect sizes, degrees of freedom and *P* value noted<br>*Give P values as exact values whenever suitable.*
☒	☐	For Bayesian analysis, information on the choice of priors and Markov chain Monte Carlo settings
☐	☒	For hierarchical and complex designs, identification of the appropriate level for tests and full reporting of outcomes
☐	☒	Estimates of effect sizes (e.g. Cohen's *d*, Pearson's *r*), indicating how they were calculated

Our web collection on statistics for biologists contains articles on many of the points above.

Software and code

Policy information about availability of computer code

Data collection	Sony SH800 software was used for flow cytometry analysis.
Data analysis	For single-cell Repli-seq (scRepli-seq) and BrdU-IP Repli-seq analyses, we used custom code from Takahashi et al., Nature Genetics (2019), and Miura et al., Nature Protocols (2020). The code is available at: https://github.com/kuzobuta/scRepliseq-Pipeline.<br><br>For RNA-seq analysis, we used the ramdaq pipeline from Hayashi et al., Nature Communications (2018), available at: https://github.com/rikenbit/ramdaq.<br><br>For haplotype-resolved and allele-specific RNA-seq analysis, we used the EMASE pipeline from Raghupathy et al., Bioinformatics (2018), available at: https://emase.readthedocs.io/en/latest/index.html.<br><br>All RNA-seq, scRNA-seq, and scRR-seq-RNA datasets were processed using a complete nextflow pipeline, ramdaq v.1.9.220. The following tools were used; Nextflow v.23.04.01, FastQC v.0.11.8, Fastq-mcf v.1.04.807, Hisat v.2.2.0, Samtools v.1.10, Bam2Wig v.3.0.1, Bamtools v.2.5.1, read_distribution v.3.0.1, inter_experiment v.3.0.1, inner_distance v.3.0.1, junction_annotation v.3.0.1, featureCounts v.2.0.1, RSEM v.1.3.1, edgeR v.3.26.5, and MultiQC v.1.9.<br><br>For DNA-seq analysis, we used the following tools: bwa (v.0.7.17-r1188), bedtools v.2.25.0-1, AneuFinder v.1.2.1, samtools v.1.3.1, samstat v.1.5.1, fastqc v.0.11.8, cutadapt v.1.18, seqtk v.1.3, picard v.2.20.2.

For manuscripts utilizing custom algorithms or software that are central to the research but not yet described in published literature, software must be made available to editors and reviewers. We strongly encourage code deposition in a community repository (e.g. GitHub). See the Nature Portfolio guidelines for submitting code & software for further information.

Data

Policy information about availability of data

All manuscripts must include a data availability statement. This statement should provide the following information, where applicable:

- Accession codes, unique identifiers, or web links for publicly available datasets
- A description of any restrictions on data availability
- For clinical datasets or third party data, please ensure that the statement adheres to our policy

All NGS datasets have been deposited in GEO under accession number GSE278959 [https://www.ncbi.nlm.nih.gov/geo/query/acc.cgi?acc=GSE278959]. We have also shared the code used to generate figures on our GitHub repository [https://github.com/mcbmieu/scRR-seq/].

Research involving human participants, their data, or biological material

Policy information about studies with human participants or human data. See also policy information about sex, gender (identity/presentation), and sexual orientation and race, ethnicity and racism.

Reporting on sex and gender	Human research is not involved in this study.
Reporting on race, ethnicity, or other socially relevant groupings	Human research is not involved in this study.
Population characteristics	Human research is not involved in this study.
Recruitment	Human research is not involved in this study.
Ethics oversight	Human research is not involved in this study.

Note that full information on the approval of the study protocol must also be provided in the manuscript.

Field-specific reporting

Please select the one below that is the best fit for your research. If you are not sure, read the appropriate sections before making your selection.

☒ Life sciences ☐ Behavioural & social sciences ☐ Ecological, evolutionary & environmental sciences

For a reference copy of the document with all sections, see nature.com/documents/nr-reporting-summary-flat.pdf

Life sciences study design

All studies must disclose on these points even when the disclosure is negative.

Sample size	No statistical method was used to predetermine sample size. The sample size was chosen based on previous experience and standards in the field. For whole-S analysis, sample sizes were selected to ensure coverage of cells at various stages throughout S phase (Takahashi et al., Nature Genetics, 2019).
Data exclusions	For scRR-seq-DNA, we first calculated the mapping ratio by dividing the number of reads with MAPQ >10 by the total number of reads. Cells with a mapping ratio below Q1 – 1.5×IQR were excluded. We also calculated the MAD score using the log2 ratio of read counts to the genome-wide median across non-overlapping 200-kb windows. We filtered out cells with MAD scores >0.3 for G1 cells, and <0.4 or >0.8 for mid-S phase cells. After sorting the cells based on their percentage replication scores, we calculated Manhattan distances between samples. Cells with a Manhattan distance greater than Q3 + 1.5×IQR were excluded. Cells with percentage replication scores below 10% or above 90% were also excluded from downstream analysis.<br><br>For scRR-seq-RNA, we first quantified the number of uniquely mapped reads to the genome (using hisat) in each cell and filtered out cells with values below Q1 – 1.5×IQR. In addition, we examined uniquely mapped reads to ribosomal RNA (rRNA) and mitochondrial RNA (mtRNA) and filtered out those with values above Q3 + 1.5×IQR for either. Cells that failed to pass the quality control criteria for either scRR-seq-DNA or scRR-seq-RNA were excluded from all subsequent analysis.
Replication	For Fig. 1, mid-S phase RPE1 cells for scRR3 were pooled from two independent biological experiments, whereas scRR1 cells were derived from one biological experiment. For Fig. 2, two mouse 8-cell embryos (biological replicates) were collected and processed in parallel. For Fig. 3a and 4a, whole-S phase RPE1 cells were pooled from two independent biological experiments each for scRR1 and scRR3. For Fig. 3b and 4d, whole-S phase CBMS1 mESCs were derived from one biological experiment and subjected to scRR1. For Fig. 5a, Mid-S phase HAP1 cells were derived from one biological experiment, and for Fig. 6, IMR-90 cells were derived from one biological experiment. For all single-cell data, multiple single-cell samples were independently processed under identical conditions, and genome-wide datasets were generated to confirm data reproducibility.
Randomization	Randomization is not relevant to this study because no comparisons between experimental groups were made.

Blinding	Blinding was not relevant to this study because all metrics were derived from absolute quantitative methods without human subjectivity.

Reporting for specific materials, systems and methods

We require information from authors about some types of materials, experimental systems and methods used in many studies. Here, indicate whether each material, system or method listed is relevant to your study. If you are not sure if a list item applies to your research, read the appropriate section before selecting a response.

Materials & experimental systems

n/a	Involved in the study
☐	☒ Antibodies
☐	☒ Eukaryotic cell lines
☒	☐ Palaeontology and archaeology
☐	☒ Animals and other organisms
☒	☐ Clinical data
☒	☐ Dual use research of concern
☒	☐ Plants

Methods

n/a	Involved in the study
☒	☐ ChIP-seq
☐	☒ Flow cytometry
☒	☐ MRI-based neuroimaging

Antibodies

Antibodies used	Anti-BrdU antibody for BrdU-IP Repli-seq profiles (dilution to 12.5 ug/ml by PBS) from BD Biosciences Pharmingen (cat. 555627)
Validation	The antibody is commercially available and have associated datasheets from the supplier. Anti-BrdU antibody was validated to immunoprecipitate BrdU-containing DNA and used for the previous study (Takahashi et al., Nature Genetics 2019).

Eukaryotic cell lines

Policy information about cell lines and Sex and Gender in Research

Cell line source(s)	Female CBMS1 mouse embryonic stem cells (Dr. Kimi Araki's lab, Kumamoto University), Human TERT-RPE1 (Clontech, C4001-1), Human HAP1 (Horizon, C859), Human IMR90 (JCRB Cell Bank, JCRB9054).
Authentication	Karyotypes of all cell lines were verified through NGS analysis.
Mycoplasma contamination	The cell lines were not tested for mycoplasma contamination.
Commonly misidentified lines (See ICLAC register)	No commonly misidentified cell lines were used.

Animals and other research organisms

Policy information about studies involving animals; ARRIVE guidelines recommended for reporting animal research, and Sex and Gender in Research

Laboratory animals	C57BL/6 mice, aged 8–12 weeks were used.
Wild animals	The study did not involve wild animals.
Reporting on sex	The study used both male and female embryos.
Field-collected samples	The study did not involve samples collected from the filed.
Ethics oversight	The animals were housed in environmentally controlled rooms, and all the experimental procedures using animals were approved by the Institutional Animal Care and Use Committee of RIKEN Kobe Branch (protocol number: A2015-06-9).

Note that full information on the approval of the study protocol must also be provided in the manuscript.

Plants

Seed stocks	No plant materials were used in this study.
Novel plant genotypes	No plant materials were used in this study.
Authentication	No plant materials were used in this study.

Flow Cytometry

Plots

Confirm that:

☒ The axis labels state the marker and fluorochrome used (e.g. CD4-FITC).

☒ The axis scales are clearly visible. Include numbers along axes only for bottom left plot of group (a 'group' is an analysis of identical markers).

☒ All plots are contour plots with outliers or pseudocolor plots.

☒ A numerical value for number of cells or percentage (with statistics) is provided.

Methodology

Sample preparation	Cultured cells were sorted and collected using a Sony SH800 cell sorter, while cells isolated from embryos were collected manually through micromanipulation.
Instrument	Sony SH800 cell sorter
Software	The data was collected by Cell Sorter Software.
Cell population abundance	Approximately 1,000,000 to 2,000,000 cells were used for cell sorting per sample, with 70–80% showing a typical cell cycle profile.
Gating strategy	An FSC/BSC gate was used for gating the population of cells to exclude cell debris. Then, a Hoechst 33342 gate was used to exclude doublet cells. The gates for sorting the G1 or desired S-phase fractions were defined on the Hoechst 33342 histogram. For single-cell collection, cells were sorted using the single-cell mode.

☒ Tick this box to confirm that a figure exemplifying the gating strategy is provided in the Supplementary Information.
